# Supplementary material for: Influenza A Virus H7 nanobody recognizes a conserved immunodominant epitope on hemagglutinin head and confers heterosubtypic protection
Source: Nat Commun. 2025 Jan 9;16:432. doi: 10.1038/s41467-024-55193-y (PMC11718266; doi:10.1038/s41467-024-55193-y)
Supplement: Supplementary file 1 — Supplementary Information [file 41467_2024_55193_MOESM1_ESM.pdf]

Supporting Information for

Influenza A Virus H7 nanobody recognizes a conserved immunodominant epitope on hemagglutinin head and confers heterosubtypic protection

Zhao-Shan Chen[1, 2], Hsiang-Chi Huang[2,3,4], Xiangkun Wang[1], Karin Schön[2], Yane Jia[1], Michael Lebens[2], Danica F. Besavilla[2], Janarthan R. Murti[2], Yanhong Ji[1], Aishe A. Sarshad[3,4], Guohua Deng[5], Qiyun Zhu[1, *], Davide Angeletti[2, 6, *]

[1]*State Key Laboratory for Animal Disease Control and Prevention, College of Veterinary Medicine, Lanzhou University, Lanzhou Veterinary Research Institute, Chinese Academy of Agricultural Sciences, Lanzhou, China*

[2]*Department of Microbiology and Immunology, Institute of Biomedicine, University of Gothenburg, Gothenburg, Sweden*

[3] *Department of Medical Biochemistry and Cell Biology, Institute of Biomedicine, University of Gothenburg, Gothenburg, Sweden*

[4] *Wallenberg Centre for Molecular and Translational Medicine, University of Gothenburg, Gothenburg, Sweden*

[5] *State Key Laboratory of Animal Disease Control and Prevention, Harbin Veterinary Research Institute, Chinese Academy of Agricultural Sciences, Heilongjiang, China*

[6] *SciLifeLab, Institute of Biomedicine, University of Gothenburg, Gothenburg, Sweden*

*Corresponding authors:

Qiyun Zhu (zhuqiyun@caas.cn)

Davide Angeletti (davide.angeletti@gu.se)

Contents of this file

Supplementary Table 1

Supplementary Figures 1-6

Supplementary Table 1. PCR primers used in this study.

Primer name	Primer sequence
**Fragment A-Forward**	ATCATTTTGGCAAAGGAATTCGAGCTCGGTACCCGG
**Fragment A-Reverse**	GAGTGTTCATTATGTTTTTGTCACCCTGCTTTTGCTCCCC
**Fragment B-Forward**	CAAAAACATAATGAACACTCAAATCCTGGTATTCGC
**Fragment B-Reverse**	TTTTTTAGTATTATATACAAATAGTGCACCGCATGTTTCCA
**Fragment C-Forward**	TTGTATATAATACTAAAAAACACCCTTGTTTCTACTAATAACCCG
**Fragment C-Reverse**	AAAAAGATCTGCTAGCTCGAGCATGCCCGGGTACCAT
**IgG-Forward**	GTCCTGGCTGCTCTTCT
**IgG-Reverse**	GGTACGTGCTGTTGA
**VHH-Forward**	CTACAAATGCCTATGCATCCCAGGTGCAGCTCGTGGAGTC.
**VHH-Reverse**	AAACAACTTTCAACAGTGGAGGGGTCTTCGCTGTGGTGCG
**pComb-Forward**	AAAGAATATCGCATTTCTTCTTGCATCT
**pComb-Reverse**	AGGAGACGGTGACCTGGGTCCCCTG
**Nb-Forward**	TCCAGTGTGGTGGAATTCGCCACCAAAAAGAATATCGCATTTCTTC TTGCATCT
**Nb-Reverse**	TCTAGACTCGAGTCAGTGATGGTGGTGGTGGTGGTGGTGGTGTGAGG AGACGGTGACCTGGG
**GAPDH-Forward**	GTCATTGAGAGCAATGCCAG
**GAPDH-Reverse**	GTGTTGCTACCCCCAATGTG
**HA-Forward**	ATGAACACTCAAATCCTGGT
**HA-Reverse**	TTATATACAAATAGTGCACC

A

Pro	2.5844	2.5349	2.2234	2.4812	2.6553	2.0399	2.1616	2.2566	2.5813	2.6464	2.2611	2.5904
	2.3277	2.5618	2.5059	2.1862	2.4761	2.5564	2.2086	2.2079	2.4225	2.4103	2.3822	2.3129
	2.2422	2.2521	2.3624	2.4614	2.1851	2.2816	2.5236	2.5039	2.5006	2.4689	2.3540	2.3173
	2.4294	2.2686	2.3537	2.4503	2.5418	2.1935	2.3901	2.2024	2.3786	2.3759	2.3634	2.7421
	2.3006	2.3595	2.4405	2.4652	2.5334	2.2572	2.4085	2.3536	2.3847	2.3429	2.2580	2.8712
	2.4748	2.4145	2.5453	2.5109	2.4904	2.4416	2.4677	2.4578	2.0823	2.5587	2.4731	2.5530
	2.4345	2.5656	2.5248	2.5474	2.8563	2.0292	2.5360	2.2703	2.6137	2.5589	2.5242	2.8171
	2.6393	2.5519	2.5447	2.5620	2.3547	2.4623	2.6422	2.4424	2.5308	2.6024	2.5028	2.8671
PVA	0.2206	0.1846	0.1691	0.2315	0.1755	0.1986	0.1734	0.1858	0.1787	0.1862	0.1491	0.1939
	0.1587	0.1247	0.1596	0.1545	0.1481	0.1659	0.1534	0.1640	0.1473	0.1510	0.1462	0.1721
	0.1922	0.1746	0.1620	0.2247	0.1767	0.1518	0.1847	0.2003	0.1627	0.1471	0.1246	0.1466
	0.1470	0.1788	0.2007	0.1523	0.1640	0.1845	0.2081	0.1709	0.1778	0.1514	0.1351	0.1590
	0.1829	0.1273	0.1594	0.1970	0.2258	0.1459	0.1816	0.1348	0.1599	0.1210	0.1287	0.1576
	0.1453	0.0997	0.1389	0.1578	0.1361	0.1691	0.1795	0.1094	0.1413	0.1308	0.1245	0.1554
	0.1891	0.1665	0.1428	0.1706	0.1864	0.1295	0.1366	0.1528	0.1337	0.1153	0.1109	0.1533
	0.1818	0.1465	0.1438	0.1604	0.1953	0.1320	0.1499	0.1294	0.1719	0.1248	0.1402	0.1478
Pro/PVA	11.7153	13.7319	13.1484	10.7179	15.1299	10.2714	12.466	12.1453	14.4449	14.2127	15.165	13.3595
	14.6673	20.5437	15.7011	14.1502	16.7191	15.4093	14.3977	13.4628	16.446	15.9623	16.2941	13.4393
	11.666	12.8986	14.5827	10.9542	12.3662	15.0303	13.6632	12.5007	15.3694	16.7838	18.8925	15.807
	16.5265	12.6879	11.7275	16.0886	15.4988	11.8889	11.4853	12.8871	13.378	15.6929	17.4937	17.2459
	12.5785	18.535	15.3105	12.5137	11.2197	15.4709	13.2627	17.4599	14.9137	19.3628	17.5447	18.2183
	17.0323	24.2177	18.3247	15.9119	18.2983	14.4388	13.7476	22.4662	14.7367	19.5619	19.8643	16.4286
	12.8741	15.409	17.6807	14.932	15.3235	15.6695	18.5652	14.858	19.549	22.1934	22.761	18.3764
	14.5176	17.4191	17.6961	15.9726	12.0568	18.6538	17.6264	18.8748	14.7225	20.8526	17.8516	19.3985

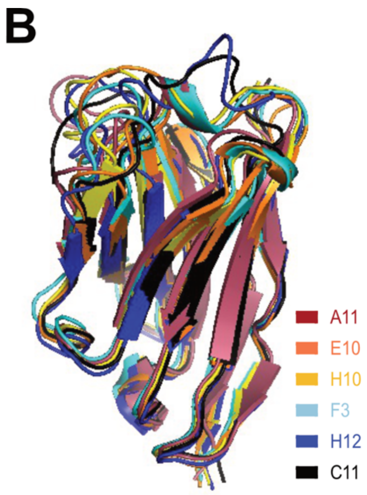

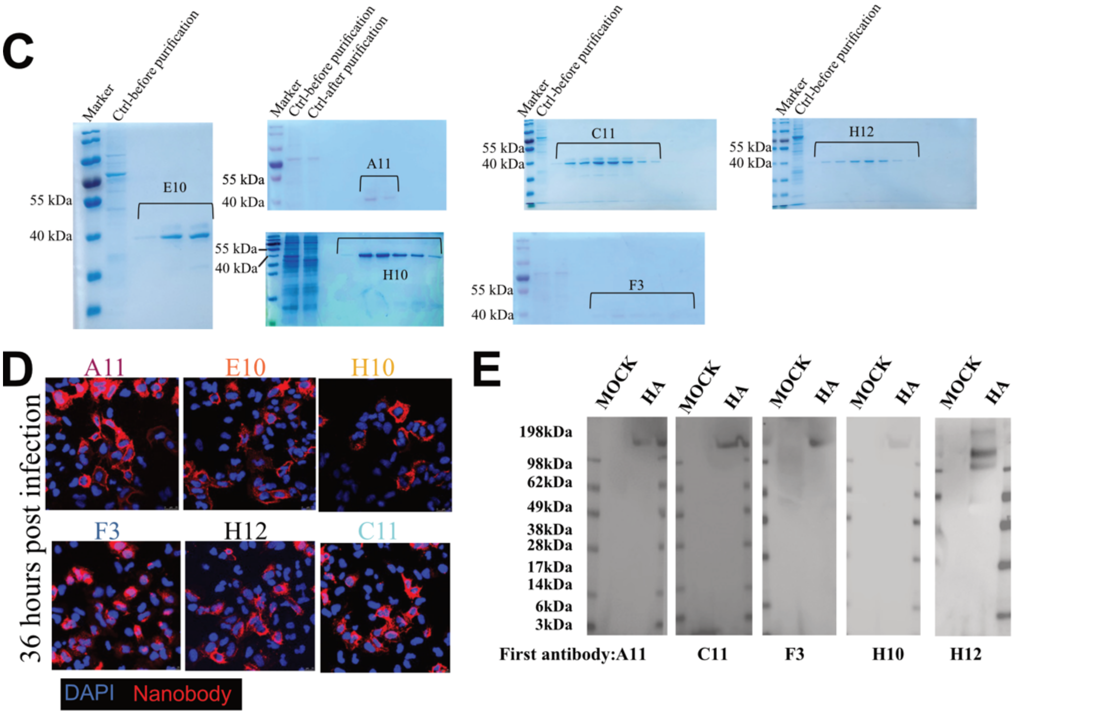

F

Sequence of nanobodies:

	FR1	CDR1	FR2	CDR2
**A11**	QVQLVESGGGLVQPGGSLRLSCAAS	GASITTYNMR	WVRQAPGKDLEWVS	QINSDGIRPWYTDSVKG
**E10**	QVQLVESGGGLVATGGSLRLSCTAS	GFSLDRYAIG	WFRQAPGKEREGIS	CISSRRDRTNYADSVNGRLT
**F3**	QVQLVESGGGLVQPGGSLRLSCAAS	KFTLDNYSIG	WFRQAPGKEREEIS	CSSARDSATYYADSVKG
**C11**	QVQLVESGGDLVQPGGSLRLTCALS	LFTTGYYTIG	WFRQAPGKEREGVS	AISYNAGSTDYSDSVKG
**H10**	QVQLVESGGGLVQPGGSLRLSCATS	GTTLDNYSIG	WFRQAPGKEHEEIS	CTSAGDGTTYYADSVKG
**H12**	QVQLVESGGGLVQPGGSLRLSCAAS	GFTLDYYAMD	YHAIGWFRQAPGKE	REEVSCTSRDGSTNYAESVKG

FR3	CDR3	FR4
RFTISRDNAKNTLYLQMNSLKPEDTAVYYCGN	GAVAGGRLLGG	WGQGTQVTVSS
VSRDNAKNTVYLQMNSLKPEDTAVYYCAA	DRSSYTYCSFRLSEYDY	WGQGTQVTVSS
RFTISRDNAKSTVYLQLNSLKPEDTAVYYCTA	AGSWYCPATPDY	WGQGTQVTVSS
RFTISRDNSKNTVYLQMNSLKPEDTAVYYCAA	KGNWYPSLSRSDYDY	WGQGTQVTVSS
RYTISRDNAKNTVYLQMNSLKPEDTAVYYCTA	AGSWYCPATPDY	WGQGTQVTVSS
RFTISRDNAKNTVYLQMNSLKSEDTAVYYCTA	AGSWYCPASPDY	WGQGTQVTVSS

Supplementary Figure 1. Generation and characterization of H7-specific nanobodies.

(A) Binding ability of 96 selected phage colonies as detected by Indirect-ELISA using H7. (20 high binding colonies: yellow). (B) Nanobodies structural model created using

ImmuneBuilder and AlphaFold 2. (Different colors represent different nanobody: A11: red, C11: black, E10: orange, F3: blue, H10: yellow, H12: dark bule.) (C) Coomassie Brilliant Blue Staining detection of the expression of different nanobodies. (D)Immunofluorescence assay (IFA) showing the recognition of the SZ19 virus by different nanobodies. A549 cells were infected with SZ19 H7 IAV (MOI=0.1) for 36h and stained with distinct primary nanobodies, followed by secondary goat anti-human IgG Fc Alexa Fluor™ 488 (A11: red, E10: orange, H10: yellow, F3: blue, H12: dark blue, C11: black.). Scale bar, 250 μm (E) Western blot analysis of A549 cells infected with SZ19 H7 IAV (MOI=1) for 24h. Cell lysate was probed with respective nanobodies and detected using goat anti-human IgG-Fc HRP. Data is representative of at least two independent experiments. (F) Sequence of six selected nanobodies.

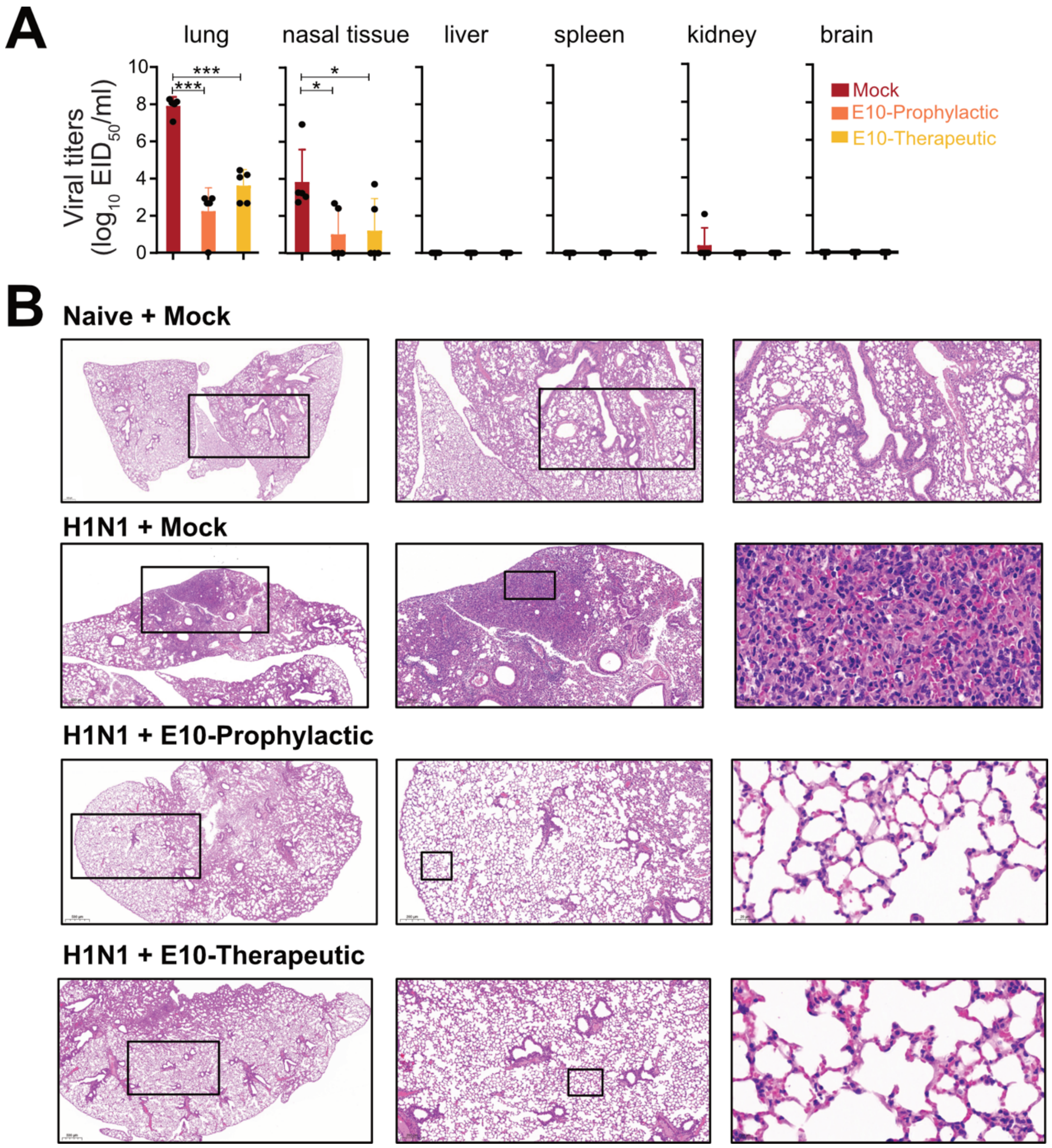

Supplementary Figure 2. E10-Fc treatment protects mice against homo- and heterosubtypic IAV challenge. (A) Viral titer, measured by EID_{50}, in six different organs (lung, nasal, liver, spleen, kidney, brain) on day 3 after H1N1 PR8 infection with or without E10 administration, as outlined in 2E. Graphs shows mean +/- SD. (B) Representative histopathological analysis of lungs from mice on day 3 after H1N1 PR8 infection with or without E10 administration, and of MOCK-infected mice, as outlined 2E. Scar bar, 500 µm in the left row, 200 µm in the middle row, 20 µm in the right row.

A

Nanobody	Escape mutants (eggs)	Escape mutants (cells)
A11	K140N	A122P, K140S
C11	-	-
F3	-	-
H10	S167L, G205E	S167L
H12		K140R, G144E, K173E

B

Nanobody	Sequences enriched (phage display)	Position
A11	VNEEALRQILRESGGIDKEA<br>RESGGIDKEAMGFTYNGIRT	113-122
C11	QFELIDNEFNEVEKQIGNVI*3	391-410
F3	AGFIENGWEGLIDGWYGFRH<br>LIDGWYGFRHQNAQGEGTAA	346-355
H10	TVTFSFNGAFIAPDRASFLR*3	241-260
H12	IERREGSDVCYPGKFVNEEA<br>YPGKFVNEEALRQILRESGG<br>VNEEALRQILRESGGIDKEA	103-107

C

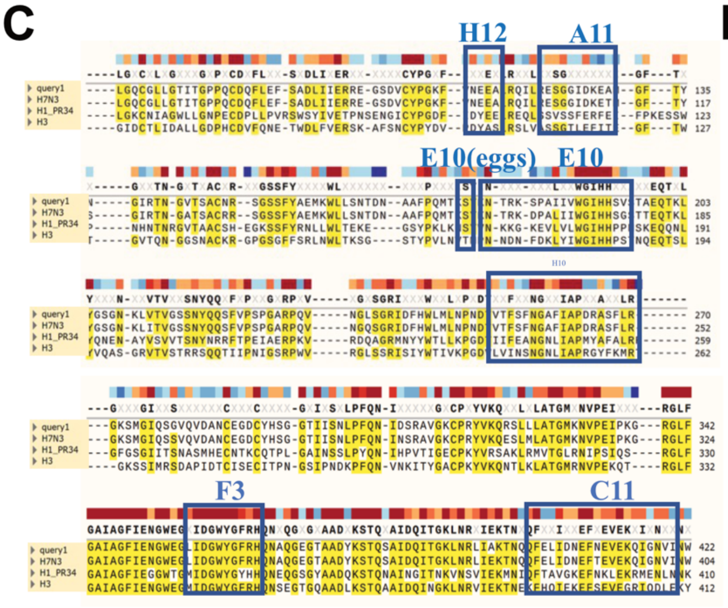

D

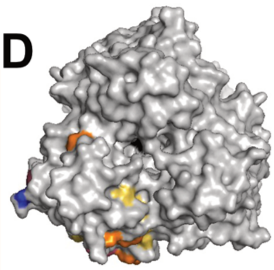

Supplementary Figure 3. E10 recognizes a conserved epitope located on HA-head lateral patch. (A) Nanobody escape mutations in the H7N9 virus were identified by selection in SPF eggs and MDCK cells. Variations at key residues involved in nanobody escape are highlighted, showing the sites where resistance developed. (B) Phage display selection of other 5 nanobody identified specific peptides. The red region depicts an overlapping area among selected peptides. (C) HA subtype numbering conversion of H7N9-SZ19- HA protein with H1N1-PR8-HA and H3N2-HA. Alignment performed on the website of NIAID Bioinformatics Resource Centers (https://www.bv-brc.org/). The blue boxes show the different nanobodies binding regions, as identified by phage display or eescape selection. (D) Epitope mapping of the nanobodies on SZ19 H7N9 HA protein showing the binding sites of six different nanobodies, each labeled with distinct colors (A11: red, E10: orange, H10: yellow, F3: blue, H12: dark blue, C11: black). Complementary view to Fig 4C. The head domain of SZ19 H7 HA was modeled using Swiss-Model. Images were generated using Open-Source PyMOL version 2.5.0.

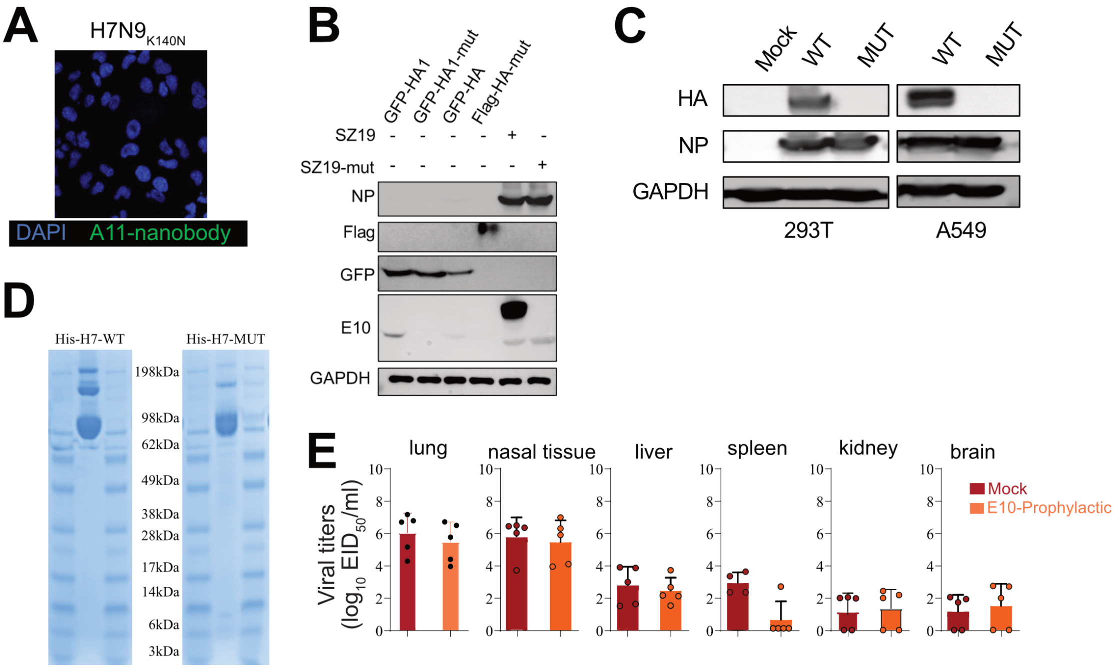

Supplementary Figure 4. H7-HA$_{K166T, S167L}$ double mutant escapes E10 recognition but has lower viral fitness. (A) IFA of A549 infected with H7N9$_{K140N}$ virus (MOI=0.1) at 24h post-infection. Cell was stained with A11 as the primary antibody, followed by secondary goat anti-human IgG Fc Alexa Fluor™ 488. Nuclei were counterstained with DAPI (blue), and NP detection is shown in green. Data is representative of at least two independent experiments. (B) Western blot (WB) analysis of A549 cells infected with WT or MUT virus or transfected with GFP-HA and GFP-HA-MUT with or without E10 pre-incubation, showing detection of NP and E10. Data are representative of at least two independent experiments. Shown are the mean values of three technical replicates. (C) Western blot (WB) showing the infection of A549 or 293T cells 24h post infection with WT and MUT virus. Data are representative of at least two independent experiments. Shown are the mean values of three technical replicates. (D) Coomassie Brilliant Blue Staining detect the express and size of recombinant WT-HA and MUT-HA protein. (E) Viral titer, measured by EID_{50}, in six organs (lungs, nasal tissue, liver, spleen, kidney, brain) on day 3 post-MUT infection, with or without E10 treatment, as described in 5E. Data are representative of three independent experiments and shown as mean values from three technical replicates. Statistical analysis was performed using unpaired t test. $^*p < 0.05$; $^{**}p < 0.01$; $^{****}p < 0.0001$; ns: not significant.

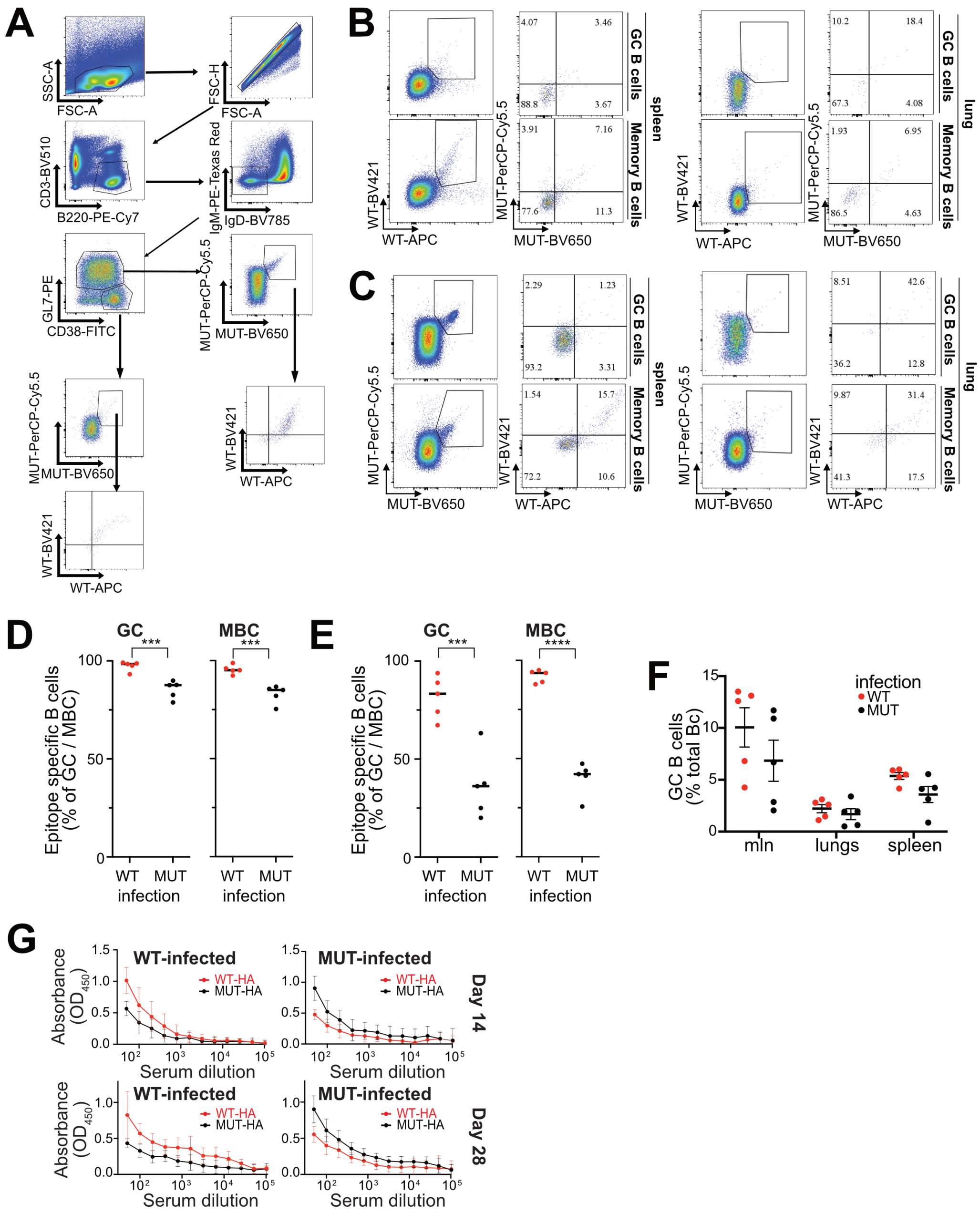

Supplementary Figure 5. E10-epitope is immunodominant upon H7-IAV infection.. (A) Flow cytometry gating for B cell characterization and analysis. (B) Representative flow cytometry gating of GC and MBC and epitope identification in spleen and lung of 14 days infected WT mice, gated GC as live CD3− B220+ IgD− IgM− GL7+ CD38− WT+ MUT+, MBC as live CD3− B220+ IgD− IgM− GL7− CD38+ WT+ MUT+. (C) Same as in B but for MUT infected mice, gated GC as live CD3− B220+ IgD− IgM− GL7+ CD38− MUT+ WT+, MBC as live CD3− B220+ IgD− IgM− GL7− CD38+ MUT+ WT+. (B, C) Quantification of epitope-specific MBC and GC B cells in lung and spleen of 14 days infected WT or MUT mice. (D, E) Percentage of HA-specific B cells at spleen/lung after infected with WT or MUT virus 14 days. Two independent experiments with 5 mice each. Bars represent SEM; statistical analysis was performed using two-sided unpaired t test. (F) Percentage of B cells at mln/spleen/lung after infected with WT or MUT virus 14 days. Two independent experiments with 5 mice

each. Bars represent SEM.(G) ELISA curves showing the detection of WT-HA and MUT-HA in serum of WT or MUT infected mice, at 14, 28 days post infection. Data represent four independent experiments with 5 mice each (n = 20). Bars represent mean ± SEM

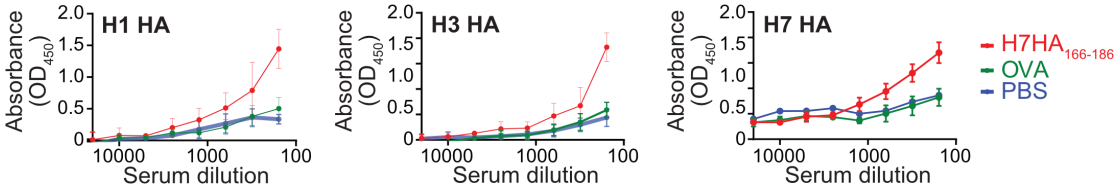

Supplementary Figure 6. H7HA$_{166\text{-}186}$ peptide immunization confers partial protection from lethal H7N9 infection. ELISA test showing binding of serum of mice immunized with H7-HA$_{166\text{-}186}$ peptide (red) or relevant controls (OVA-immunized mice: green; PBS immunized mice: blue) to H1N1/H3N2/H7N9 HA proteins. Representative of two independent experiments with 4-5 mice per group. Bars represent mean ± SEM
