# Supplementary material for: Influenza A Virus H7 nanobody recognizes a conserved immunodominant epitope on hemagglutinin head and confers heterosubtypic protection
Source: Nat Commun. 2025 Jan 9;16:432. doi: 10.1038/s41467-024-55193-y (PMC11718266; doi:10.1038/s41467-024-55193-y)
Supplement: Supplementary file 2 — Reporting summary [file 41467_2024_55193_MOESM2_ESM.pdf]

nature portfolio

Corresponding author(s): Davide Angeletti, Qiyun Zhu

Last updated by author(s): Nov 18, 2024

Reporting Summary

Nature Portfolio wishes to improve the reproducibility of the work that we publish. This form provides structure for consistency and transparency in reporting. For further information on Nature Portfolio policies, see our Editorial Policies and the Editorial Policy Checklist.

Statistics

For all statistical analyses, confirm that the following items are present in the figure legend, table legend, main text, or Methods section.

n/a	Confirmed	
☐	☒	The exact sample size (*n*) for each experimental group/condition, given as a discrete number and unit of measurement
☐	☒	A statement on whether measurements were taken from distinct samples or whether the same sample was measured repeatedly
☐	☒	The statistical test(s) used AND whether they are one- or two-sided<br>*Only common tests should be described solely by name; describe more complex techniques in the Methods section.*
☒	☐	A description of all covariates tested
☐	☒	A description of any assumptions or corrections, such as tests of normality and adjustment for multiple comparisons
☐	☒	A full description of the statistical parameters including central tendency (e.g. means) or other basic estimates (e.g. regression coefficient) AND variation (e.g. standard deviation) or associated estimates of uncertainty (e.g. confidence intervals)
☐	☒	For null hypothesis testing, the test statistic (e.g. *F*, *t*, *r*) with confidence intervals, effect sizes, degrees of freedom and *P* value noted<br>*Give P values as exact values whenever suitable.*
☒	☐	For Bayesian analysis, information on the choice of priors and Markov chain Monte Carlo settings
☒	☐	For hierarchical and complex designs, identification of the appropriate level for tests and full reporting of outcomes
☒	☐	Estimates of effect sizes (e.g. Cohen's *d*, Pearson's *r*), indicating how they were calculated

Our web collection on statistics for biologists contains articles on many of the points above.

Software and code

Policy information about availability of computer code

Data collection	Leica TCS SP8 multi-photon microscope was used to collect the confocal fluorescence signal.<br>BD LSR II Flow Cytometer for flow cytometry.<br>CTL ImmunoSpot plate readerwere used for Real Time PCR signal collection.<br>ÄKTA pure chromatography system were used for purify nanobody.<br>CTL ImmunoSpot plate reader were used for ELISPOT plate reading.<br>TECAN Sunrise absorbance microplate reader (catalog: 16039400) were used for ELISA testing.

Data analysis	FlowJo V10 software was used for FACS data analyzing.<br>All statistical analysis and P values were obtained using the GraphPad Prism software version 7.0 (GraphPad Software, Inc. USA).<br>Open-Source PyMOL version 2.5.0. (http://www.pymol.org) was used for proein visualization.<br>Global Initiative on Sharing All Influenza Data (GISAID) (https://www.gisaid.org/) database and the Influenza Virus Database of GenBank (https://www.ncbi.nlm.nih.gov/genomes/FLU/Database/nph-select.cgi?go_database) were used to download HA sequences.<br>ImmuneBuilder(https://github.com/oxpig/ImmuneBuilder) and AlphaFold 3.0 (https://alphafoldserver.com/about) were used to stimulate nanobody structures.<br>Swiss-model (https://swissmodel.expasy.org/) were used to model the structure of SZ19-HA.<br>CaseViewer software was used for HA strain (https://www.3dhistech.com/solutions/caseviewer/).<br>The ClusPro 2.0 server was used in "antibody mode" to produce docking models between nanobody E10-Fc and SZ19 H7-HA. The top 30 models returned by the server were ranked depending on energy and cluster size. The 10 best ClusPro docking models were further analyzed based on interactions and interface properties calculated according to PDBePISA (Proteins, Interfaces, Structures and Assemblies) (Europen Bioinformatics Institute (https://www.ebi.ac.uk/pdbe/prot_int/pistart.html))

For manuscripts utilizing custom algorithms or software that are central to the research but not yet described in published literature, software must be made available to editors and reviewers. We strongly encourage code deposition in a community repository (e.g. GitHub). See the Nature Portfolio guidelines for submitting code & software for further information.

Data

Policy information about availability of data

All manuscripts must include a data availability statement. This statement should provide the following information, where applicable:

- Accession codes, unique identifiers, or web links for publicly available datasets
- A description of any restrictions on data availability
- For clinical datasets or third party data, please ensure that the statement adheres to our policy

All data presented in the manuscript is available from the corresponding author upon reasonable request

Research involving human participants, their data, or biological material

Policy information about studies with human participants or human data. See also policy information about sex, gender (identity/presentation), and sexual orientation and race, ethnicity and racism.

Reporting on sex and gender	N/A
Reporting on race, ethnicity, or other socially relevant groupings	N/A
Population characteristics	N/A
Recruitment	N/A
Ethics oversight	N/A

Note that full information on the approval of the study protocol must also be provided in the manuscript.

Field-specific reporting

Please select the one below that is the best fit for your research. If you are not sure, read the appropriate sections before making your selection.

☒ Life sciences ☐ Behavioural & social sciences ☐ Ecological, evolutionary & environmental sciences

For a reference copy of the document with all sections, see nature.com/documents/nr-reporting-summary-flat.pdf

Life sciences study design

All studies must disclose on these points even when the disclosure is negative.

Sample size	No statical methods were used to predetermine sample size. Same size were chosen based on previous experience for each experiments to provide sufficient sample number for common statistical test including t test and two way ANOVA used in this study. Experiments were performed with three samples per condition unless started otherwise. Animal sample size was at least four mice and determined by experimental feasibility and sample availability to demonstrate certain results. The exact number of sample for each experiments was noted in the associated figure legend. Statistical methods were not used to predetermine sample size.
Data exclusions	No data exclusion.
Replication	All replication attempts were successful. Experiments were independently repeated at least two to three times with similar results.

Randomization	Animals were randomly allocated to each group as described in the form below. All mice were Females
Blinding	The investigators were not blinded regarding to the groups

Reporting for specific materials, systems and methods

We require information from authors about some types of materials, experimental systems and methods used in many studies. Here, indicate whether each material, system or method listed is relevant to your study. If you are not sure if a list item applies to your research, read the appropriate section before selecting a response.

Materials & experimental systems

n/a	Involved in the study
☐	☒ Antibodies
☐	☒ Eukaryotic cell lines
☒	☐ Palaeontology and archaeology
☐	☒ Animals and other organisms
☒	☐ Clinical data
☒	☐ Dual use research of concern
☒	☐ Plants

Methods

n/a	Involved in the study
☒	☐ ChIP-seq
☐	☒ Flow cytometry
☒	☐ MRI-based neuroimaging

Antibodies

Antibodies used

anti-Human IgG (Fc specific)-488, Invitrogen, catalog: A55747, 1:200 for IFA.
DAPI, Beyotime, catalog: C1002 for IFA.
anti-GAPDH , abcm, catalog: 181602, 1:5000 for WB.
horse anti-mouse IgG , Vector laboratories, catalog: ZK0403, 1:1000 for WB.
Mouse Anti-Human IgG Fc Antibody, Genscript, catalog: 50B4A9, 1:1000 for WB.
anti-mouse Igκ, Sino Biological, catalog: 68077-R008-H, 1:5000 for ELISA.
Mouse Anti-Human IgG Fc Antibody, Sino Biological, catalog: SSA001, 1:6000, for ELSIA and WB.
anti-mouse CD3 BV510, BD, catalog: 563024,1:700 for FACS.
anti-mouse IgM PE/Dazzle 594, BioLegend, catalog: 314529, 1:200 for FACS.
anti-mouse IgD BV785, BD, catalog: 563618,1:200 for FACS.
anti-mouse CD38 FITC, BioLegend, catalog: 102705,1:200 for FACS.
anti-mouse GL7 PE, BioLegend, catalog: 144607,1:200 for FACS.
anti-mouse B220 Pe-Cy7, BioLegend, catalog: 103221,1:200 for FACS.
streptavidin-APC, Bioligand, catalog: 405243, According to the instruction manual for FACS.
Brilliant Violet 421 Streptavidin, BioLegend, catalog:405225, According to the instruction manual for FACS.
Brilliant Violet 650 Streptavidin, BD, catalog:563855, According to the instruction manual for FACS.
Live/Dead Aqua, Invitrogen, catalog: L34966, According to the instruction manual for FACS.
IgG H+L HRP, Aviva Systems Biology, catalog: OAIA00330, 1:5000 for ELISPOT.
Percp5.5 Streptavidin (BioLegend, catalog:405214),According to the instruction manual for FACS.

Validation

All antibodies used in this work were purchased from companies, and validated by the manufacturers and by extensive use in published work. All western blot, FACS, ELISA, IFA antibodies in the manuscript had been validated. Each antibody has been validated for the spaces and application on the manufacturer's website.
Antibodies used in WB were validated in cell:
anti-GAPDH (abcm, 181602, https://www.abcam.com/en-us/products/primary-antibodies/gapdh-antibody-epr16891-loading-control-ab181602), horse anti-mouse IgG (Vector laboratories, ZK0403, https://vectorlabs.com/products/peroxidase-horse-anti-mouse-iggsrsltid=AfmBOooV9LdjujCjJ8Wmggi0b9GYZE5GUXcyI4JYcYCLHSto3PEZ93u), Mouse Anti-Human IgG Fc Antibody (Genscript, 50B4A9, https://www.genscript.com/antibody/A01854-Mouse_Anti_Human_IgG_Fc_Antibody_HRP_mAb.html), Mouse Anti-Human IgG Fc Antibody (Sino Biological, catalog: SSA001, https://www.sinobiological.com/antibodies/secondary-antibody-goat-anti-human-igg-fc-ssa001),
Antibodies used in ELISA were validated in mice:
anti-mouse Igκ (Sino Biological, 68077-R008-H, https://www.sinobiological.com/antibodies/human-igkc-loc500183-68077-r008),
Antibodies used in IFA were validated in cells: DAPI, (Beyotime, C1002, https://www.beyotime.com/product/C1002.htm)
Antibodies used in IFA were validated in human:
anti-Human IgG (Fc specific)-488 (Invitrogen, A55747, https://www.thermofisher.com/antibody/product/Goat-anti-Human-IgG-Fc-Secondary-Antibody-clone-3H8L9-Recombinant-Monoclonal/A55747),
Antibodies used in ELISPOT were validated in mice:
IgG H+L HRP (Aviva Systems Biology, OAIA00330, https://www.avivasysbio.com/human-igg-h-l-antibody-hrp-conjugated-oaia00330.html?srsltid=AfmBOormL3kKyoH42chL9cIOlNLr88WP0HciiEHAH_vgO-n8UpVJFiRh)
Antibodies used in FACS were validated in mice:
anti-mouse CD3 BV510 (BD, catalog: 563024,https://www.bdbiosciences.com/en-se/products/reagents/flow-cytometry-reagents/research-reagents/single-color-antibodies-ruo/bv510-hamster-anti-mouse-cd3e.563024), anti-mouse IgM PE/Dazzle 594, (BioLegend, catalog: 314529, https://www.biolegend.com/fr-ch/products/pe-dazzle-594-anti-human-igm-antibody-12466), anti-mouse IgD BV785, BioLegend, 563618,https://www.bdbiosciences.com/en-eu/products/reagents/flow-cytometry-reagents/research-reagents/single-color-antibodies-ruo/bv786-rat-anti-mouse-igd.563618),anti-mouse CD38 FITC, (BioLegend, 102705,https://www.biolegend.com/en-gb/products/fitc-anti-mouse-cd38-antibody-182),

anti-mouse GL7 PE (BioLegend, 44607,https://www.biolegend.com/de-at/products/pe-anti-mouse-human-gl7-antigen-t-and-b-cell-activation-marker-antibody-9122?GroupID=BLG11093),anti-mouse B220 Pe-Cy7 (BioLegend, 03221,https://www.biolegend.com/ja-jp/products/pe-cyanine7-anti-mouse-human-cd45r-b220-antibody-1930), streptavidin-APC (Bioligand, 405243, https://www.biolegend.com/fr-lu/products/apc-streptavidin-high-concentration-10081?GroupID=GROUP23), Brilliant Violet 421 Streptavidin(BioLegend, 405225,https://www.biolegend.com/en-gb/products/brilliant-violet-421-streptavidin-7297?GroupID=GROUP23), Brilliant Violet 650 Streptavidin (BD, 563855, https://www.bdbiosciences.com/en-se/products/reagents/flow-cytometry-reagents/research-reagents/single-color-antibodies-ruo/bv650-streptavidin.563855), Percp5.5 Streptavidin (BioLegend, 405214, https://www.biolegend.com/de-de/products/percp-cyanine5-5-streptavidin-4212)

Eukaryotic cell lines

Policy information about cell lines and Sex and Gender in Research

Cell line source(s)	293T cells, A549, 293F and MDCK cells were purchased from ATCC (Maryland, USA), MDCK-SIAT1 were gifted by Dr.Jonathan Yewdell at NIH.
Authentication	The cell lines were not authenticated
Mycoplasma contamination	The cells tested negative for mycoplasma
Commonly misidentified lines (See ICLAC register)	*Name any commonly misidentified cell lines used in the study and provide a rationale for their use.*

Animals and other research organisms

Policy information about studies involving animals; ARRIVE guidelines recommended for reporting animal research, and Sex and Gender in Research

Laboratory animals	Mice, Female C57BL/6 mice, aged 8-12 weeks, were purchased from Janvier, France, and housed in a specific pathogen-free (SPF) facility at f the Experimental Biomedicine Unit, University of Gothenburg. Additional female C57BL/6 mice infected with H7N9 vieus were obtained from SPF (Beijing) Biotechnology Co., Ltd. in China and housed under similar conditions in the biosafety level 3 laboratory in Harbin as mentioned in paper. Alpaca, male, aged 2.5 years, were provided by a local farm in Gansu Province, China.
Wild animals	No wild animals were used in this study.
Reporting on sex	Only female mice were used in the study
Field-collected samples	N/A
Ethics oversight	All experimental procedures were approved by the Animal Care and Use Committee of Experimental Biomedicine Unit, University of Gothenburg. The protocol was approved by the Committee on the Ethics of Animal Experiments of the Harbin Veterinary Research Institute (HVRI) of the Chinese Academy of Agricultural Sciences (CAAS). All mice were housed in isolated ventilated cages (large one maxima 10 mice,small one maxima 5 mice) barrier facility at gothenburg university. The mice were maintained on a 12/12-hour light/dark cycle,22-26 degree with sterile pellet food and water ad libitum and 40%-70% humidity, similar in CAAS.

Note that full information on the approval of the study protocol must also be provided in the manuscript.

Plants

Seed stocks	*Report on the source of all seed stocks or other plant material used. If applicable, state the seed stock centre and catalogue number. If plant specimens were collected from the field, describe the collection location, date and sampling procedures.*
Novel plant genotypes	*Describe the methods by which all novel plant genotypes were produced. This includes those generated by transgenic approaches, gene editing, chemical/radiation-based mutagenesis and hybridization. For transgenic lines, describe the transformation method, the number of independent lines analyzed and the generation upon which experiments were performed. For gene-edited lines, describe the editor used, the endogenous sequence targeted for editing, the targeting guide RNA sequence (if applicable) and how the editor was applied.*
Authentication	*Describe any authentication procedures for each seed stock used or novel genotype generated. Describe any experiments used to assess the effect of a mutation and, where applicable, how potential secondary effects (e.g. second site T-DNA insertions, mosiacism, off-target gene editing) were examined.*

Flow Cytometry

Plots

Confirm that:

☒ The axis labels state the marker and fluorochrome used (e.g. CD4-FITC).

☒ The axis scales are clearly visible. Include numbers along axes only for bottom left plot of group (a 'group' is an analysis of identical markers).

☒ All plots are contour plots with outliers or pseudocolor plots.

☒ A numerical value for number of cells or percentage (with statistics) is provided.

Methodology

Sample preparation	Mouse GC B cell and MBC were isolated from spleen,mln and lung. Cells were cultured in FACS buffer in DPBS as described in Methods.<br>For B cell characterization, antibody mixture was added to each sample and incubated for 30 minutes at 4°C . The samples were then washed and incubated for 30 minutes at 4°C. To exclude dead cells, Live/Dead Aqua (Invitrogen, catalog: L34966) staining was performed, followed by fixation of the cells with 1.5% paraformaldehyde (PFA). After fixation, the samples were washed, resuspended in 200µl of FACS buffer, and stored at 4°C until analysis. All details are in the Methods.
Instrument	BD LSR II Flow Cytometer
Software	FACS Diva software (BD) version 8.0
Cell population abundance	N/A
Gating strategy	Cells were first gated using FSC/SSC characteristics to exclude debris,followed by gating FSC-A and FSC-H, then SSC-A and SSC-H to eliminate non-singlets.<br>Then target cells were gated the population of interest by specific staining.<br>The details of the gating strategy was provided in the Supplementary information.

☒ Tick this box to confirm that a figure exemplifying the gating strategy is provided in the Supplementary Information.
